# The factors associated with the knowledge of brushing teeth with fluoridated toothpaste among high school students in Al-Madinah, Saudi Arabia

**DOI:** 10.3389/froh.2024.1416718

**Published:** 2024-06-20

**Authors:** Saba Kassim, Alla T. Alsharif

**Affiliations:** Department of Preventive Dental Sciences, College of Dentistry, Taibah University, Al-Madinah Al-Munawwarah, Saudi Arabia

**Keywords:** adolescence, awareness, brushing, teeth, fluoride

## Abstract

**Objective:**

The use of fluoridated toothpaste (FT) is essential for controlling caries. This analytical cross-sectional study aimed to determine the proportion of students who brushed their teeth with fluoridated toothpaste/or do not know the content at least once a day and to determine the factors associated with the knowledge of brushing teeth with FT.

**Methods:**

An anonymous questionnaire was distributed during the academic year 2019–2020 among 439 high school students. The data collected included sociodemographic characteristics and oral-health–related variables [e.g., brushing teeth, knowledge of the effect of fluoride on caries (KEFC) and dental service utilisation (DSU)]. The dependent variable was the knowledge of using FT when brushing teeth (Yes or do not know). Descriptive, bivariate, and logistic regression analysis were performed.

**Results:**

The response rate was 98% (*n* = 432) and usable data was 88% (*n* = 385). The median (IQR) age of the students was 16.00 (1) years, and 190 (47%) were males. Eighty eight percent of the students brushed their teeth with toothpaste daily with no knowledge of toothpaste content and only 86 (21.8%) knew the content of the toothpaste used for brushing their teeth i.e., FT. The multivariable analyses revealed an association of family income and KEFC with brushing teeth with FT [adjusted odds ratio (AOR): 1.98, 95% confidence interval (CI): 1.14–3.43, *p* = 0.015 and AOR = 6.11, 95% CI: 3.45–10.83, *p* < 0.001, respectively].

**Conclusions:**

While the brushing and use of toothpaste among high school students was common, the knowledge of the content of toothpaste used for brushing teeth was less common and was associated with family income and KEFC.

## 1 Introduction

Dental services are devoted to preventing dental caries in children and adolescents as they are more cost-effective than treatments (1). Adolescence marks the phase of the complete development of permanent teeth. This period is also ideal for safeguarding oral health by practising proper dental habits and receiving preventive care for oral diseases. When dental caries are highly prevalent and severe, even modest prevention activities can result in considerable reductions in disease levels. Extensive empirical evidence suggests that fluoride plays a significant role in reducing dental caries (2–6). Water fluoridation schemes have been the cornerstone of caries-prevention strategies for caries over 5 decades (7, 8) leading to a substantial decline in dental caries rates, namely in Western countries. In recent years, increased attention has been paid to the proper use of fluoride-based interventions, highlighting its primary role in topical effects (9–11). Although various fluoride vehicles are available in different forms (drinking water, salt, milk, varnish etc.), fluoridated toothpaste is the most widely used method and remains a benchmark intervention for maintaining a constant low level of fluoride in the oral environment (12, 13).

Individuals' use of fluoridated toothpastes largely depends on their socio-cultural integration of such toothpastes into their oral hygiene habits, the availability of fluoride toothpastes and their ability to be purchased and used regularly (14). Fluoride intake is also driven by various factors, including dental care access, dentist recommendations, parental influence, education level (15) personal dental habits, age and community programmes (16). Individuals' socioeconomic status is also a potential risk indicator for children's fluoride intake levels (17). In terms of health knowledge uptake and application in health decision-making and health behaviour action, high literacy indicates having skills for gaining knowledge and applying it to health-related decisions and behaviours (18). Adolescents increasingly take on great responsibility for their well-being, particularly when making choices about their lifestyle behaviours (19). Adolescents’ view of health literacy as a type of cultural health asset that individuals utilise to participate in health-related activities plays a vital role in shaping habits (encompassing passive health literacy usage) and conscious actions (such as active health literacy engagement) in the context of health-related decision-making (20).

A recent multi-country online survey collected data from caregivers of children (most aged 6–12 years old) reported that 60.3% brushed teeth with fluoridated toothpaste (21). Yet, there is knowledge gap with respect of adolescents (14–18 years old) themselves knowledge of using floriated toothpaste when brushing teeth. Notably, adolescents are required to be prepared for the tangible adult tasks ahead of them (22). Therefore, adolescents' involvement in oral health, encompassing regular dental visits and informed toothpaste choices, is pivotal. Dental check-ups detect issues early, instilling lifelong care habits. Selecting a suitable toothpaste empowers autonomy and tailors' care. Ingraining these practices fosters responsibility and confidence, forming the basis for enduring oral well-being. Notably, the twice daily tooth brushing with fluoridated toothpaste is recommended for all dentate children as cost effective and clinically effective in reducing caries (13, 23, 24). In addition, Adolescents' knowledge of the intake of fluoridated toothpaste when brushing teeth is essential for the two following reasons: (1) to instil the importance of the use of fluoride as an important way for controlling caries and (2) for the removal and disruption of the biofilm of the disease (25).

This study aimed to report the proportion of adolescents, who will be referred to as high school students onword, who brushed their teeth with fluoridated toothpaste/or do not know the content at least once a day and to determine the factors associated with the knowledge of brushing teeth with fluoridated toothpaste among high school children in Al-Madinah, Saudi Arabia. It was hypothesised that children are not knowledgeable about the content of toothpaste used for brushing their teeth (whether fluoridate or not) regardless of their sociodemographic characteristics and knowledge of the effects of fluoride on caries.

## 2 Materials and methods

### 2.1 Study design, population, and setting

**Figure 1:**
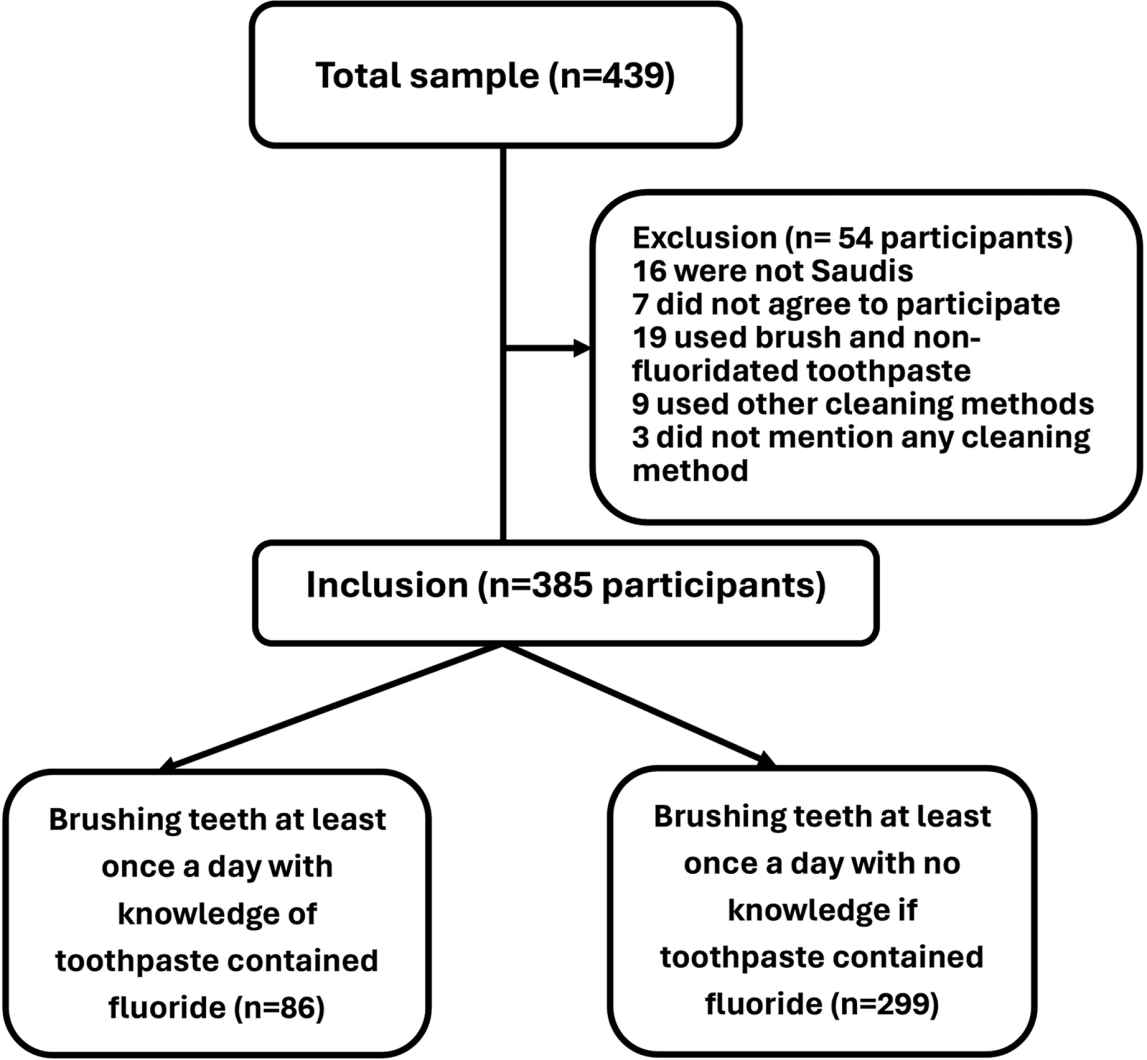
Flowchart of inclusion and exclusion criteria for high school students brushing teeth with fluoridated and do not know the content of toothpaste.

This outreach analytical cross-sectional survey was conducted during the academic year 2019–2020, before the COVID lockdown, by the Taibah University, Department of Preventive Dental Sciences. The data collection was part of the graduate students' required work in Dental Public Health and included providing oral hygiene instructions, a demonstration of the correct method of brushing teeth and nutrition advice. A convenience sample of (439) students was recruited from four high schools in the city of Al-Madinah, Kingdom of Saudi Arabia (KSA): 104 students from a private school and 335 students from three public schools. The schools and participating students were recruited based on the schools' consent. As a result, the final sample included 385 students who met the aims of this study. These children are those who clean their teeth with a toothbrush and toothpaste (fluoridated and do not know if it was fluoridated). The flowchart below demonstrates the inclusion and exclusion criteria (Figure 1).

### 2.2 Ethical considerations

The Research Ethics Committee of Taibah University, College of Dentistry, approved (Ref. TUCDRED/20200328/HABakeer) this survey. School consent was obtained as a practice in KSA. The survey adhered to the Declaration of Helsinki, i.e., the schools were informed about the purpose of the survey, their right to confidentiality and the withdrawal from the survey without giving reasons. Nevertheless, students who opted out of the survey still have the right to receive oral health promotion awareness as the mission of the College of Dentistry is to establish a community partnership and provide oral health services. The obtained data were stored on a password-protected laptop, and only the Preventive Department and its team had access to the database.

### 2.3 Data collection procedures and variables

The survey was conducted in one day only during a scheduled school time. The participating students were escorted to a room where an online form was set up on iPads. The form can be accessed through a direct link to a Google Form via the software application HyperText Markup Language (HTML) and using a valid self-administered Arabic questionnaire. The students were asked to fill in the questionnaire, which took approximately 10 min and was preceded by an information sheet about the survey in addition to electronic consent. Those who agreed to participate could fill in the questionnaire; otherwise, the questionnaire would not open. Upon completion of the questionnaire, the participants were instructed to submit the web form which was stored in an Excel spreadsheet.

The questionnaire consisted of sociodemographic variables [age in years, gender (male, female, other), parents' educational attainment (postgraduate, university degree, high school, intermediate school, primary school, illiterate), family income (>SR 50,000, SR 40,001–50,000, SR 30,001–40,000, SR 20,001–30,000, SR 10,000–20,000 and others) and type of school attended to (private or public)] and oral health variables (daily toothbrushing frequencies [once a day, two times a day, three times or more a day or do not brush teeth]), brushing teeth with fluoridated toothpaste [yes, no or do not know the content of the toothpaste], use of other cleaning methods [e.g., miswak; yes or no], dental attendance [every three months, every six months, once a year, when necessary or I do not go] (26), dental treatment payment [publicly funded, insurance, out of pocket] and knowledge of the effects of fluoride on caries (KEFC) [yes or no]. Categorical variables were recategorised due to a small number in certain categories as well as to ease data interpretation (Table 1, Results section).

**Table 1:** Sociodemographic characteristics and oral-health–related behaviours of the sample (*n* = 385).

Variables	M (IQR) or F (%)
Age, median (IQR)	16.0 (1)
Gender	
Males	182 (47.3)
Females	203 (52.7)
Type of school	
Public	90 (23.4)
Private	295 (76.6)
High school level	
First year	192 (49.9)
Second year	117 (30.4)
Third year	76 (19.7)
Father's level of education	
Less than university	134 (34.8)
University or higher	251 (65.2)
Mother's level of education	
Less than university	90 (23.4)
University or higher	295 (76.6)
Family income	
≤SR 20,000	220 (57.1)
>SR 20,000	165 (42.9)
Brushing teeth with fluoridated toothpaste	
Do not know the content of toothpaste	299 (77.7)
Yes	86 (22.3)
Dental service utilisation	
When in pain or never used	292 (75.8)
Every 3–6 months or yearly	93 (24.2)
Dental treatment payment^a^	
Out-of-pocket money	204 (54.0)
Publicly funded or insurance	174 (46.0)
KEFC	
No	276 (71.7)
Yes	109 (28.3)

M (IQR), median with interquartile range; F (%), frequencies with percentages.

KEFC, knowledge of the effects of fluoride on caries.

^a^ Valid percentage (7 missing).

### 2.4 Statistical analysis

The Excel spreadsheet was downloaded and imported to the Statistical Package for Social Sciences (SPSS software for Windows, version 21, IBM) for data processing and analysis. Descriptive statistics were performed to report sample characteristics. Categorical data were reported as frequencies and percentages and the continuous variable (age) as the median and interquartile range (IQR) because the data did not adhere to normality tests (Shapiro–Wilk was <0.05). Bivariate analyses (the *χ*^2^ and Mann–Whitney *U*-tests) were conducted to assess the association of sociodemographic and oral health related variables (e.g., dental attendance) with the frequency of brushing of teeth at least once a day with fluoridated toothpaste (yes or do not know the content of the toothpaste). Then, multivariable logistic regression analysis was performed to determine the abovementioned association. The latter analysis was presented as an adjusted odds ratio (AOR), with a corresponding 95% confidence interval (CI).

As age and family income had a stronger influence on the outcome being studied compared to both the student's grade level and the type of school they attended and to eliminate multicollinearity, students' high school level (year 1, 2 or 3) and the type of school (private or public) were excluded from the association analysis., Confounding factors, such as mother's and father's level of education, were forced into the model. As *a priori* sample size was not calculated for this survey, *post hoc* sample calculation showed sufficient power for performing the logistic regression modelling (*R*^2 ^= 0.230, predictors = 7, *p* ≤ 0.05 and observed statistical power = 1.0). *p* ≤ 0.05 was considered significant for all analyses. The SROBE guidelines for the reporting of this cross-sectional study was followed (27).

## 3 Results

Of the total sample 432 responded to participate (response rate 98%) and the usable data for the study aim (brushing teeth with fluoridated toothpaste [Yes or do not know the content of toothpaste]) was 385 (88%). As shown in Table 1, among the students who brushed their teeth with toothpaste, 47% were male and 76.6% of them studied in private schools. However, only 22.3% knew that they used fluoridated toothpaste and 28% know about the effects of fluoride on caries.

After controlling for the confounding variables, the multivariable regression modelling (Table 2) yielded two variables that are statistically significantly associated with brushing teeth with fluoridated toothpaste. First, the students who knew the effects of fluoride on caries (KEFC) were 6.11 (95% CI: 3.45–10.83, *p* < 0.001) more likely to brush their teeth with fluoridated toothpaste compared to those who did not know. This variable was the strongest predictor as indicated by the Wald value (Table 2). Second, students with family income >SR 20,000 were approximately two times more likely to brush their teeth with fluoridated toothpaste (AOR: 1.98, 95% CI: 1.14–3.43, *p* = 0.015).

**Table 2:** Results of the bivariate analysis and multivariable logistic regression analyses predicting the likelihood of self-reporting the knowledge of brushing teeth with fluoridated toothpaste among high school students in Al-Madinah, KSA, (*n* = 385; academic year 2019–2020).

Variable	Brushing with fluoridated toothpaste*			Regression modelling			
	Do not know	Yes	*p*-value	B	Wald	AOR (95% CI)	*p*-value
Age, median (IQR)	16.0	17.0	**0** **.** **005**	0.234	2.866	1.28 (0.96–1.69)	0.090
Gender			0.103				0.318
Male	148 (81.3)	34 (18.7)		−0.300	0.977	Ref	
Female	152 (74.4)	52 (25.6)				0.74 (0.41–1.34)	
Father's level of education			0.595				0.806
Less than university	102 (76.1)	32 (23.9)		0.071	0.061	Ref	
University or higher	197 (78.5)	54 (21.5)				1.07 (0.61–1.89)	
Mother's level of education			0.750				0.870
Less than university	71 (78.9)	19 (21.1)		−0.054	0.027	Ref	
University or higher	228 (77.3)	67 (22.7)				0.95 (0.50–1.81)	
Family income			**0** **.** **024**				**0** **.** **015**
≤20,000 SR	180 (81.8)	40 (18.2)	** **	0.681	5.905	Ref	
>20,000 SR	119 (72.1)	46 (27.9)	** **			1.98 (1.14–3.43)	
Dental service utilisation			**0** **.** **003**				0.249
When in pain or never used	237 (81.2)	55 (18.8)		0.348	1.330	Ref	
Every 3–6 months or yearly	62 (66.7)	31 (33.3)				1.42 (0.78–2.56)	
Dental treatment payment			0.105				0.587
Out-of-pocket money	151 (74.0)	53 (26.0)		−0.152	0.295	Ref	
Publicly funded or insurance	141 (81.0)	33 (19.0)				0.86 (0.50–1.49)	
KEFC			**<0** **.** **001**				**<0** **.** **001**
No	242 (87.7)	34 (12.3)		1.811	38.489	Ref	
Yes	57 (52.3)	52 (47.7)				6.11 (3.45–10.83)	

AOR, adjusted odds ratio; CI, confidence interval; KEFC, knowledge of the effects of fluoride on caries.

* *χ*^2^ and Mann–Whitney *U*-tests.

Bold values signify *p* ≤ 0.05.

## 4 Discussion

In Saudia Arabia approximately 70% of permanent teeth of Saudi Arabian children are affected by caries, with an average DMFT score of 3.5 (28). As reported by Al Dosari et al. caries prevalence varied between 59% and 80% among Saudi children aged 15–18, correlated with fluoride levels in the region (29). Therefore, this study assessed Saudi high school children's knowledge of the toothpaste content [fluoridated toothpaste (FT) or do not know] used when brushing their teeth at least once a day, understanding of fluoride's impact on dental caries, dental attendance, dental treatment affordability and determinants linked to the knowledge of using FT. This study refuted the hypothesis as children's knowledge of the type of toothpaste they use for brushing their teeth (fluoridated or do not know) was significantly influenced by their sociodemographic characteristics and KEFC.

The study found that a significant percentage of the respondents were unaware of the composition of their toothpaste, do not visit the dentist regularly, except when they experience discomfort, and do not know the role of fluoride in preventing dental caries. These findings were consistent with other studies indicating that most children do not know about the role of fluoride in strengthening the teeth and preventing dental caries (30–35). The outcomes of the multivariable regression analysis revealed two variables that exhibit statistically significant association with the practice of brushing teeth with FT. High school students' understanding of fluoride's effect on dental caries emerged as the first noteworthy determinant. Similar findings were reported in other studies (30, 32). This finding underscores the pivotal role of education and awareness in shaping and encouraging sound oral hygiene practices. Students who understand the benefits of fluoride in preventing dental caries are inclined towards the utilisation of fluoridated toothpaste (36). Considering these findings, comprehensive dental health education programmes that not only emphasise the significance of fluoride but also strive to increase students' knowledge and awareness must be promoted.

Additionally, family income had a significant influence. This outcome implies that socioeconomic factors exert a pivotal role in determining the likelihood of adolescents using fluoridated toothpastes. *High school students from higher-income households may possess superior access to dental care knowledge, resources, and products, including fluoridated toothpastes.* Conversely, those from lower-income backgrounds may encounter barriers to quality dental care, manifesting disparities in oral hygiene practices. Extensive research has established a robust connection between parents' socioeconomic status and oral health outcomes as well as the health literacy levels of their children (17, 37–39). These studies consistently demonstrate that a family's economic circumstances play a pivotal role in shaping the oral health (e.g., brushing teeth with fluoridated toothpaste) and health literacy of the younger generation. Addressing socioeconomic disparities in oral health through targeted interventions and policies is paramount. Through these measures, equitable access to dental care resources can be ensured across all income strata, leading to improved oral hygiene practices, and reduced dental caries prevalence among children. Notably, enhancing awareness among Saudi high school students necessitates the implementation of comprehensive nationwide campaigns. Such campaigns should incorporate educational content on oral hygiene practices, including the utilisation of fluoridated toothpaste. Integrating this vital information into the school curriculum of high school students is imperative. Additionally, the influential role of electronic media, particularly social media platforms, should be emphasised, given their significant impact on health education, and fostering changes in beliefs. Leveraging social networking sites can also be an effective strategy for reaching younger generations. Furthermore, active engagement of primary healthcare professionals is crucial in the dissemination of health education. This survey had several limitations as follows: the study employed a cross-sectional convenience sample as such this sampling method may introduce bias due to its inability to ensure the representativeness of the broader high school students population in the region and establish causal or temporal relationships between variables. The study heavily relied on self-reported data from the participating students, which could be susceptible to recall and social desirability biases and reporting inaccuracies. Generalising the study's findings beyond the specific geographic and demographic characteristics of the study population may be limited and the teeth brushing as once a day could be considered as infrequent. Despite efforts to control for confounding factors, such as parents' education level, the presence of unmeasured confounders not considered in the analysis could potentially impact the observed associations. The study has number of strengths that included high response rate, use of validated questions and collection of data in natural setting, i.e., schools rather than health care centers. Based on this survey findings and limitations, the brushing and use of toothpaste among high school students was common. However, a significant proportion of the respondents exhibit a lack of knowledge regarding the composition of their toothpaste. Notably, family income and KEFC serve as predictive factors for high schools' oral health literacy, specifically regarding their awareness of using fluoridated toothpaste.

## Data Availability

The raw data supporting the conclusions of this article will be made available by the authors, without undue reservation.
